# The efficacy of postoperative radiotherapy in localized primary soft tissue sarcoma treated with conservative surgery

**DOI:** 10.1186/s13014-016-0605-y

**Published:** 2016-02-25

**Authors:** Ru-Ping Zhao, Xiao-Li Yu, Zhen Zhang, Li-Juan Jia, Yan Feng, Zhao-Zhi Yang, Xing-Xing Chen, Jian Wang, Sheng-Lin Ma, Xiao-Mao Guo

**Affiliations:** Department of Radiation Oncology, Hangzhou Cancer Hospital, 34 Yan Guan Lane, Hangzhou, Zhejiang China; Department of Radiation Oncology, Cancer Hospital of Fudan University, 270 Dong An Road, Xuhui Shanghai, China; Department of Oncology, Shanghai Medical College of Fudan University, 270 Dong An Road, Xuhui Shanghai, China; Department of medical Oncology, Shandong Binzhou Central Hospital, 108 Southern Huancheng Road, Binzhou, Shandong China; Department of Pathology, Cancer Hospital of Fudan University, 270 Dong An Road, Xuhui Shanghai, China; Department of Radiation Oncology, Hangzhou First People’s Hospital, 261 Huan Sha Road, Hangzhou, Zhejiang China

**Keywords:** Soft tissue sarcoma, Surgery, Radiotherapy, Local recurrence, Overall survival

## Abstract

**Background:**

To evaluate the efficacy of postoperative radiotherapy (RT) on local failure-free survival (LFFS), distant metastasis-free survival (DMFS) and overall survival (OS) in patients with localized primary soft tissue sarcoma (STS) and to identify prognostic factors.

**Methods and materials:**

Between January 2000 and July 2010, 220 consecutive patients with localized primary STS, who received conservative surgery with or without postoperative RT, were enrolled in the study. Survival curves were constructed by the Kaplan-Meier method and log-rank test was used to assess statistical significance. Multivariate analysis was applied to identify the prognostic factors.

**Results:**

After a median follow-up of 68 months (range, 5–127 months), the 5-year LFFS, DMFS and OS were 70.0, 78.2 and 71.2 %, respectively. Tumor size, histological subtypes, margin status and postoperative RT were independent predictors for OS. Postoperative RT was associated with a significant reduced local recurrence risk versus surgery alone (hazard ratio [HR] = 0.408, 95 % confidence interval [CI] 0.235–0.707, *P* = 0.001), with 5-year LFFS of 81.1 and 63.6 %, respectively (log-rank, *P* = 0.004). The log-rank test showed that postoperative RT had a tendency of improving OS compared with surgery alone, with 5-year OS of 74.8 and 65.0 %, respectively (*P* = 0.089). Multivariate analysis demonstrated that postoperative RT significantly reduced mortality rate compared with surgery alone (HR = 0.512, 95 % CI 0.296–0.886, *p* = 0.017), especially in patients with liposarcoma (*p* = 0.034).

**Conclusion:**

Postoperative radiotherapy reduce both local recurrence and STS mortality in patients with localized primary STS. The efficacy of RT on survival warrants further prospective study.

**Electronic supplementary material:**

The online version of this article (doi:10.1186/s13014-016-0605-y) contains supplementary material, which is available to authorized users.

## Background

Soft tissue sarcomas (STS) are a heterogeneous group of uncommon neoplasms arising from mesenchymal tissues, accounting for less than 1 % of all malignancies [1]. The conservative surgery is the most important primary treatment for patients with STS [2]. Close margin and even positive margin may be needed to avoid amputation or preserve critical neurovascular structures, which lead to high risk of local failure in the surgical bed. The data from previous studies showed the local recurrence rate after conservative surgery alone was up to 30 % [3, 4]. Randomized trials demonstrated that the addition of postoperative radiotherapy (RT) after conservative surgery could further reduce local recurrence when compared with surgery alone [5, 6]. Based on these studies, conservative surgery plus RT became the mainstay treatment modality in patients with STS.

Although randomized trials showed postoperative RT significantly improved local control, the local control benefit associated with RT did not translate into a survival advantage. Even after 17 years of follow-up, Beane JD et al. only demonstrated a trend toward improved survival in the RT group that failed to reach statistical significance. The authors acknowledged that their study was underpowered to demonstrate a survival advantage of <20 % [7]. This scenario has been replicated in several retrospective series [8–10]. It was initially believed that the improved local control by postoperative RT could only enhance local control, while had no efficacy on survival in patients with breast cancer. After accumulating the patients who treated with RT, The reports of the early Breast Cancer Trialists’ Collaborative Group (EBCTCG) meta-analysis demonstrated that postoperative RT reduced the incidence of breast cancer recurrence and mortality [11]. It is likely that previous studies were underpowered to definitively demonstrate a survival advantage with the use of RT in patients with STS. Recently, several SEER analyses and retrospective studies that including a large number of STS reported that postoperative RT could also improve the survival of STS [12–15]. The question still remains if the improved local control with the use of RT can convert to survival benefit or not. The impacts of RT on patients with STS merit further investigation.

This paper summarized our experience with homogenous population of adult patients with STS, who were treated by the conservative modality, either surgery alone or surgery plus RT. The aim of the study is to evaluate the impacts of RT on local failure-free survival (LFFS) and distant metastasis-free survival (DMFS) and overall survival. This study also evaluates the relationship between various clinicopathologic factors and disease outcomes to identify the prognostic factors.

## Methods/materials

### Patients

We retrospectively reviewed 251 consecutive adult patients with localized primary STS during the period of January 2000 to July 2010. We excluded those patients with secondary sarcoma (*n* = 6), who received amputation or the treatment for palliative purpose (*n* = 8), surgically unresectable disease at presentation (*n* = 5), and those without follow-up data (*n* = 12). The remaining 220 patients were represented in this study. Histological diagnosis was confirmed in each case through review of the slides by a pathologist. The Institutional Review Board of Cancer Hospital of Fudan University approved review of data for this investigation.

The French Federation of Cancer Centers grading system was used for tumor grading, which was determined as low- (Grade I), intermediate- (Grade II) or high-grade (Grade III) [16]. A negative margin was defined as the absence of tumor at the inked margin. We reviewed the medical records, including operative and pathologic reports, and recorded the following information: age, gender, tumor anatomic location, histopathological subtype, size, grade, surgical margins status, lymph node status, treatment modality and toxicities.

### Treatment

All patients were reviewed at an oncology multidisciplinary board and underwent appropriate structure- and function-preserving surgical resection. In patients with the tumor adjacent to neurovascular bundles or critical structures, an attempt was made to obtain gross tumor free margin. Some patients received postoperative radiation and/or chemotherapy (generally doxorubicin and ifosfamide) based on prognostic factors predicting higher risk of local recurrence and distant metastasis. Radiotherapy was delivered through 8MV linear accelerator photon to the target volume by two opposed fields, in a schedule of 2 Gy/fraction, five fractions per week. An initial dose of 50Gy was delivered after surgical wound healing (within 8 weeks after surgery). Field borders were either the whole compartment, or proximal and distal margins of 5 cm in non-compartmental lesions and trunk. A cone-down field of the original tumor bed plus 3 cm margins received an additional boost (10–20Gy). Few patients did not receive full dose RT for protecting organs at risk adjacent radiation field.

### Follow-up

The items of follow-up included physical examination, chest CT and ultrasonography of the primary site, with additional MRI when local recurrence was suspected. The patients were followed at 3-month interval for the first 2 years, 6-month interval for the following 3 years and yearly thereafter. The major endpoints of this study were local recurrence, distant metastasis and overall survival. Local recurrence was defined as the first pathological verified tumor of the same histological type, within or contiguous to the previously treated tumor bed at least 3 months after treatment. Distant metastasis was defined by clinical, pathological or radiologic evidence of systemic disease spread outside the primary tumor site.

### Toxicity

Toxicities were evaluated by chart review using the Common Terminology Criteria for Adverse Events (CTCAE) version 3.0. Only wound complications were recorded in patients received surgery alone. The radiation-related toxicities were recorded in patients received RT. The highest grade of any observed toxicities reported for each patient at the time of follow-up. Only ≥grade 2 toxicities were reported.

### Statistical analysis

Descriptive data was compared using Pearson Chi-square test or Fisher’s exact test, where appropriate. For survival analysis, survival curves were constructed using the Kaplan-Meier method. The effect of demographic, clinical, pathologic and treatment variables on survival were examined using the log-rank test to assess statistical difference. Multivariate analyses were performed using Cox logistic regression method and *P*-value of <0.01 was included into multivariate analysis to fully identify prognostic factors. A two-sided *P*-value of <0.05 determined statistical significance for all tests. All statistical analyses were performed using SPSS 17.0 software package.

## Results

### Patient characteristics

Patient, tumor, and treatment characteristics of study population were listed in Table 1. The median age at diagnosis was 50 years (range, 18–86 years) and 60 % of patients were male. Exactly half tumors (110 cases, 50.0 %) were located in extremities, 52 (23.6 %) in trunk, 45 (20.5 %) in retroperitoneum and 13 (5.9 %) in head/neck region. The most common histological subtype was liposarcoma (41.4 %). The median tumor size was 7 cm (range, 2–42 cm). RT was administered to 81 patients (36.8 %). The radiation dose ranged from 45 Gy to 70 Gy, with a median dose of 60 Gy.

**Table 1:** Patient, tumor and treatment characteristics (*n* = 240)

Characteristic	Number	Percent
Age (years)		
≤50	114	51.8
>50	106	48.2
Gender		
Male	132	60.0
Female	88	40.0
Location		
Extremity	110	50.0
Trunk	52	23.6
Head/neck	45	20.5
Retroperitoneum	13	5.9
Histology		
Rhabdomyosarcoma	27	12.3
Fibrosarcoma	23	10.5
MFH	54	24.5
Liposarcoma	90	40.9
Others	26	11.8
Tumor size (cm)		
≤5	80	36.4
>5	140	63.6
Grade		
I	31	14.1
II	58	26.4
III	131	59.5
Margin status		
Negative	195	88.6
Positive	25	11.4
Lymph node status		
Negative	210	95.5
Positive	10	4.5
Chemotherapy		
No	179	81.4
Yes	41	18.6
Radiotherapy		
No	139	63.2
Yes	81	36.8

The patients were divided into RT group and no RT group according to whether they received RT or not. The distribution of the patient characteristics was listed in Table 2. There were higher proportion of patients with tumors located in the extremities and head/neck, MFH, grade III, positive margin and the usage of chemotherapy in RT group, while the no RT group had a higher rate of patients with tumors located in retroperitoneum, liposarcoma, grade I and negative margin. The distribution of other clinicopathological factors was not significantly different between two cohorts (*P* >0.05).

**Table 2:** Distribution of patient, tumor and treatment characteristics by receiving RT or not

	RT		No RT		
Characteristic	No.	%	No.	%	*P* Value*
Age (years)					0.786
≤50	41	50.6	73	52.5	
>50	40	49.4	66	45.5	
Gender					0.967
Male	49	60.5	83	59.7	
Female	32	39.5	56	40.3	
Location					
Extremity	53	65.4	57	41.0	0.001
Trunk	15	18.5	37	26.6	0.191
Head/neck	11	13.6	2	1.4	<0.001
Retroperitoneum	2	2.5	43	30.9	<0.001
Histology					
Rhabdomyosarcoma	12	14.8	15	10.8	0.400
Fibrosarcoma	8	9.9	15	10.8	0.831
MFH	28	34.6	26	18.7	0.010
Liposarcoma	22	27.2	68	48.9	0.002
Others	11	13.6	15	10.8	0.525
Tumor size (cm)					0.773
≤5	30	37.0	50	36.0	
>5	51	63.0	89	64.0	
Grade					
I	4	4.9	27	19.4	0.002
II	21	25.9	37	26.6	1.000
III	56	69.1	75	54.0	0.033
Margin status					0.029
Negative	66	81.5	128	92.1	
Positive	15	18.5	11	7.9	
Lymph node status					0.177
Negative	75	92.6	135	97.1	
Positive	6	7.4	4	2.9	
Chemotherapy					0.019
No	59	72.5	120	86.3	
Yes	22	27.2	19	13.7	

*Abbreviation*: *RT* radiotherapy

*Determined by chi-square test

### Endpoints and survival analysis

#### Local failure-free survival

**Fig. 1:**
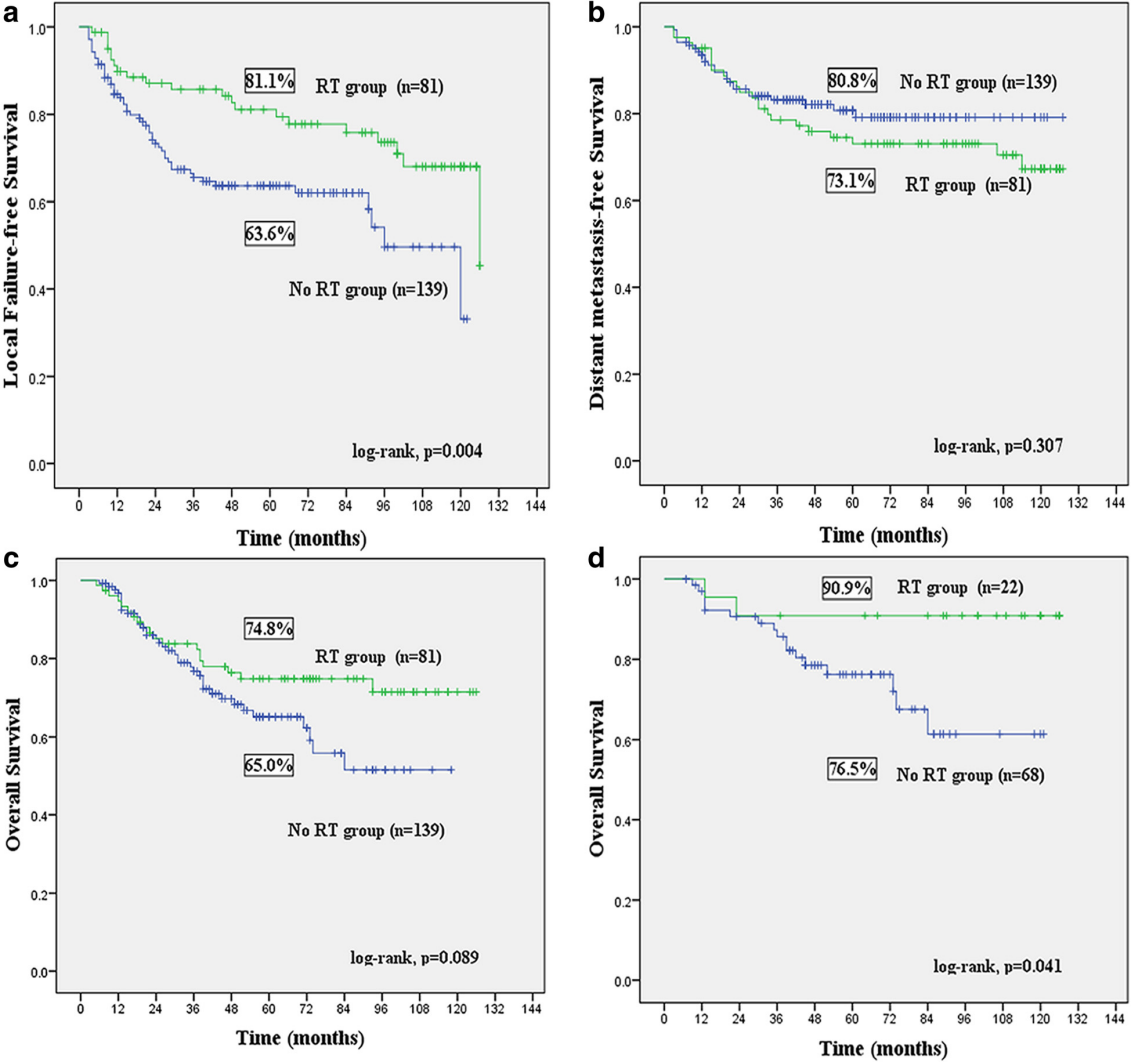
Kaplan-Meier estimates comparing patients receiving RT (RT group) and without receiving RT (No RT group) for **a** local failure-free survival; **b** diatant metastasis-free survival; **c** overall survival; **d** overall survival in liposarcoma

With a median follow-up of 68 months (range, 5–127 months), a total of 72 patients experienced local recurrence. Fifty-three patients had only local recurrence as their initial site of failure, while in three patients, local recurrence and distant metastases were detected concurrently. Of 53 patients with local recurrence as initial site of treatment failure, the median time to local failure was 23 months (range, 3–126 months). The 5-year LFFS rate was 70.0 %. In the multivariate analysis for LFFS, tumor size, margin status and postoperative RT emerged as independent prognostic factors (*p* <0.05). Postoperative RT significantly improved local control compared with surgery alone [hazard ratio (HR) = 0.408, 95 % confidence interval (CI) 0.235–0.707, *P* = 0.001, Table 3], with 5-year LFFS of 81.1 and 63.6 %, respectively (log-rank, *P* = 0.004, Fig. 1a). Larger tumor size (HR = 1.815, 95 % CI 1.051–3.138 *p* = 0.033) and positive margin (HR = 2.595, 95 % CI 1.432–4.702 *p* = 0.002) correlated with worse outcome. The other clinicopathological factors were not significant in model.

**Table 3:** Multivariate analysis for LFFS, DMFS and OS

	LFFS		DMFS		OS	
Characteristic	HR (95 % CI)	P*	HR (95 % CI)	*p*	HR (95 % CI)	
Age (>50 vs. ≤50)		0.980		0.774		0.210
Gender		0.602		0.780		0.794
Location		0.560		0.453		0.506
Histology		0.621		<0.001		<0.001
Rhabdomyosarcoma			1.00		1.00	
Fibrosarcoma			0.128 (0.029–0.569)	0.012	0.142 (0.039–0.510)	0.003
MFH			0.574 (0.281–1.174)	0.088	0.516 (0.254–1.047)	0.067
Liposarcoma			0.161 (0.069–0.378)	<0.001	0.186 (0.088–0.392)	<0.001
Others			0.407 (0.154–1.074)	0.074	0.426 (0.169–1.073)	0.070
Tumor size (cm)		0.033		0.066		0.003
≤5	1.00				1.00	
>5	1.815 (1051–3.138)				2.638 (1.392–4.998)	
Grade		0.147		0.259		0.400
Margin status		0.002		0.143		<0.001
Negative	1.00		1.00		1.00	
Positive	2.595 (1.432–4.702)		2.187 (1.055–4.536)		3.942 (2.233–6.959)	
Lymph node status		0.909		0.272		0.582
Chemotherapy		0.687		0.894		0.719
Radiotherapy		0.001		0.769		0.017
No	1.00				1.00	
Yes	0.408 (0.235–0.707)				0.512 (0.296–0.886)	

*Abbreviations*: *HR* hazard ratio, *CI* confidence interval, *OS* overall survival, *LFFS* local failure-free survival, *DMFS* distant metastasis-free survival

*Determined by Cox logistic regression method

#### Distant metastasis-free survival

Forty-eight patients developed distant metastases in this study, including 30 patients with distant metastases only and 18 patients with distant metastases and local recurrence as well. Of the 30 patients with distant metastases as initial site of treatment failure, the median time to distant metastasis was 15 months (range, 3–114 months). The 5-year DMFS rate was 78.2 %. The multivariate analysis showed that the significant predictor for distant metastasis was only histological subtype. Rhabdomyosarcoma correlated with worst outcome, while fibrosarcoma and liposarcoma predicted for better DMFS, with HR of 0.148 (95 % CI 0.033–0.661, *p* = 0.012) and 0.126 (95 % CI 0.056–0.303, *p* <0.001, Table 3), respectively. Although univariate analysis showed grade, lymph node status and chemotherapy were significant predictors for distant metastasis, these factors lost significant in the following Cox logistic regression analysis (Additional file 1: Table S1).

#### Overall survival

A total of 65 deaths occurred during follow-up, including 60 patients who died of sarcoma-related causes. Other causes of death included stroke (one patient), respiratory failure (one), heart failure (one) and second cancers (two). Of the 60 patients who died of sarcoma-related causes, 19 patients developed local recurrence only, 18 patients developed distant metastases only and 23 patients developed both local recurrence and distant metastases. The 5-year OS rate was 71.2 %. The univariate analysis showed RT had a tendency of improving OS, with 5-year OS of 74.8 and 65.0 % in the RT group and no RT group, respectively (*P* = 0.089, Fig. 1c). The multivariate analysis demonstrated that RT was associated with improved OS (HR = 0.512, 95 % CI 0.296–0.886, *p* = 0.017, Table 3). In the multivariate analysis, tumor size >5 cm was also an independent significant adverse prognostic factor compared to tumor size ≤5 cm, with a HR of 2.638 (95 % CI 1.392–4.998, *p* = 0.003). Additional adverse prognostic factor was positive margin status (HR = 3.942, 95 % CI 2.233–6.959, *p* <0.001). Rhabdomyosarcoma was the worst histological subtype. Comparison with rhabdomyosarcoma, patients with liposarcoma (HR = 0.186, 95 % CI 0.088–0.392, *p* <0.001) and fibrosarcoma (HR = 0.142, 95 % CI 0.039–0.510, *p* = 0.003) were associated with significant better survival.

### The subgroup analysis on radiation effect

Local recurrence was developed in 21 of 81 patients (25.9 %) who received RT and 51 of 139 patients (36.7 %) who did not received RT, respectively. In RT group, 13 patients developed local recurrence alone, 15 patients developed distant metastases alone, and eight patients developed both local and distant failure. In no RT group, 40 patients developed local failure alone, 14 patients developed distant metastases alone and 11 patients experienced both local and distant failure. Postoperative RT significantly improved local control compared with no postoperative RT (HR = 0.408, 95 % CI 0.235–0.707, *P* = 0.001, Table 3), with a 5-year actuarial LFFS of 81.1 and 63.6 %, respectively (log-rank, *P* =0.004, Fig. 1a). The median time to local recurrence in RT group and no RT group were 29 months (range, 4–126 months) and 15 months (range, 3–120 months), respectively. The log-rank test showed that RT postponed the time to local recurrence in patients who experienced local recurrence (*P* = 0.022). RT had no impact on the risk of development distant metastasis, with 5-year DMFS of 73.1 and 80.8 % in the RT group and no RT group, respectively (*P* = 0.307, Fig. 1b).

When we examined the impact of RT on OS, we noted a tendency of improving OS, with 5-year OS of 74.8 and 65.0 % in the RT group and no RT group, respectively (*P* = 0.089 Fig. 1c). The multivariate analysis demonstrated that RT was associated with improved OS (HR = 0.512, 95 % CI, 0.296–0.886, *p* = 0.017, Table 3). Only two patients with retroperitoneal STS received RT in our study, which might bias the final survival results. We excluded these patients with retroperitoneal STS and re-analyzed the efficacy of RT on OS. Multivariate analysis confirmed that RT improved the OS (HR = 0.450, 95 % CI, 0.243–0.833, *P* = 0.011). We also performed univariate analyses to identify specific subgroups of patients that derived benefit from RT (Additional file 1: Table S2). The result showed that RT significantly improved the 5-year OS in the subset of patients with liposarcoma (90.9 % vs. 76.5 %, *p* = 0.041, Fig. 1d).

### Toxicity

Sixteen of 139 patients (11.5 %) in the no RT group compared to 13 of 81 patients (16.0 %) in the RT group had ≥grade 2 wound complications. In the patients received RT, 31 (38.3 %) patients suffered from ≥grade 2 radiation dermatitis. Late ≥grade 2 radiation-related toxicities were recorded including peripheral nerve damage, edema, joint stiffness and bone fracture. One (1.2 %) patient developed radiation-related peripheral nerve damage, 10 (12.3 %) patients had edema, 8 (9.9 %) patients suffered from joint stiffness and bone fractures were not seen.

## Discussions

Our result showed that postoperative RT significantly reduced the risk of local recurrence, while had no impact on development of distant metastasis. When we examined the impact of postoperative RT on OS, we noted RT had a tendency of improving OS. Multivariate analysis demonstrated that RT was a significant prognostic factor for OS.

Randomized trials had demonstrated that postoperative radiotherapy could improve local control in patients with STS when compared to surgery alone, especially in patients with high grade tumor. Yang et al. showed that postoperative RT significantly decreased the 10-year local recurrence rate among patients with high-grade lesions (no local recurrence in the RT group Vs 22 % in no RT group, *p* = 0.0028) [6]. In another study, Adjuvant brachytherapy improves local control after complete resection of soft tissue sarcomas in patients with high-grade tumors, with the 5-year local control rate were 89 and 66 % in the RT and no RT group(*p* = 0.0025), respectively [5]. The data from other retrospective studies also demonstrated that RT could improve local control in patients with STS [8–10, 17, 18]. In our study, the effect of RT on local control was in line with previous reports. Although the impact of radiation on local control for extremity sarcoma has been well studied, the effects of local control on survival are less well understood.

Multiple variety studies have examined the question of survival benefit for postoperative RT in STS. However, few of those studies had sufficiently power to detect a survival advantage. Recently, several studies observed that RT improved the survival of STS. In a study including 6960 patients with STS, Koshy et al. reported that the 3-year overall survival rate was 73 % in the radiation group compared with 63 % in the no radiation group (*p* <0.001). RT was associated with a significantly improved OS (HR = 0.78, 95 % CI, 0.69–0.89) [12]. Schreiber et al. analyzed a cohort of 983 patients with high-grade sarcoma. The authors found a survival benefit at 3 years for patients with high-grade tumors >5 cm treated with radiotherapy (73.4 vs. 55.6 %, *P* <0.001) [19]. Kachare et al. found that RT was associated with a 5 % 5-year survival advantage in a study including 2606 patients with high-grade sarcoma of the extremity. The authors concluded radiotherapy, regardless of the timing, was associated with improved survival in high-risk sarcoma [15]. In another study, Gutierrez et al. observed a statistically significant increase in median overall survival of 3 months in patients undergoing RT (*p* <0.001). Administration of RT for high-grade lesions increased median survival to 25 months compared to only 16 months when RT was not used (*p* <0.001) [20]. Alkis et al. found postoperative RT was associated with significant improved OS in a retrospective study including 294 patients with STS, with 5-year OS were 62.2 % and 32.1 % (*P* <0.0001) in patients who received postoperative RT and surgery alone, respectively [13]. Another retrospective analysis of 202 patients with high grade STS of the extremity, brachytherapy following limb-sparing surgery resulted in favorable 5-year local control and OS rates [14]. All the previous studies indicated RT could improve the survival of patients with STS, which was in line with our results.

The scenario of RT improves survival can be interpreted as the improvement of local control by RT translates into survival benefit. In fact, the relationship between local control and prognosis of STS has long been debated. There were many published studies showed that patients with local recurrence had worse prognosis. The rationale may be illustrated as follow. Firstly, RT may prevent further tumor seeding by reducing local recurrence. Lewis et al. analyzed the correlation of local recurrence with subsequent metastases in 911 patients with extremity STS. The metastasis after local recurrence significantly increased in patients with high-grade and deep tumors. They concluded that there was a strong association of local recurrence with the development of subsequent metastasis and tumor mortality [21]. Secondly, although the majority patients with STS died of distant metastasis, local recurrence can also directly influence survival. Gronchi et al. observed nearly 20 % of patients with STS receiving R1 resection died of loco-regional recurrences without developing distant metastases [22]. Lewis et al. reported 84 of 112 (75 %) patients with primary retroperitoneal STS died in the absence of distant metastasis. The local recurrence rather than distant metastasis was the primary cause of death [23]. A study from M. D. Anderson Cancer Center (MDACC) reported that a significant fraction of patients who died of STS (46 of 372 patients, 12 %) had only local recurrence. The authors believed that local recurrence was likely to influence disease specific survival as well [24]. In our study, of 60 patients who died of cancer-related causes, 19 patients (32 %) died of local recurrence without developing distant metastasis. The improvement of local control by using of RT has potential take survival benefit in those patients.

Sequencing of radiotherapy may also affect outcomes in patients with STS. The only phase III randomized study comparing preoperative to postoperative RT was conducted by O’Sullivan et al. [25]. One hundred and ninety patients were stratified by tumor size and randomized to preoperative (94 patients) or postoperative RT (96 patients). Patients who had preoperative RT had more wound complications than those receiving postoperative RT after treatment (35 % vs. 17 %, *P* = 0.01). However, at a median follow-up of 6.9 years, patients treated with preoperative RT had a lower frequent of subcutaneous fibrosis (31.5 % vs. 48.2 %, *P* = 0.07), joint stiffness (17.8 % vs. 23.2 %, *P* = 0.51), and edema (15.1 % vs. 23.2 %, *P* = 0.26). There was no difference in terms of survival or local, regional, and distant failure rates between the two delivery methods [26]. Since acute wound complications can be managed and are generally temporary whereas late toxicities are longer lasting, preoperative RT is increasingly favored.

Histology, size, margin status and RT were identified as significant prognostic factors for OS in our study, which was generally in line with other reports. Stojadinovic et al. identified that tumor size, grade, and resection margin were significantly associated with sarcoma specific survival in 2123 patients with completely resected localized primary STS [27]. Pisters et al. reported that tumor size, grade, the histology, margin status and location were the prognostic factors for disease specific survival [28]. Zagars et al. studied the prognostic factors for disease specific survival in patients with localized STS treated with conservative surgery and RT. They found that the independent factors that affected disease specific survival were tumor grade, tumor size, tumor site, histopathology, patient age, and resection margins [24]. Maretty-Nielsen et al. established that age, size, grade, margin, and radiotherapy were important prognostic factors for both local recurrence and disease specific mortality in a cohort study of 922 consecutive patients with STS [29].

## Conclusion

STS is a disease largely limited to retrospective reports given the paucity incidence. It is hard to study this disease systematically and therefore little information was provided to assess the long-term benefits of RT. The current study presented the outcomes of a single institution analysis of 220 patients with localized primary STS treated by conservative strategy. Our study showed that RT reduced local recurrence and had potential effect to improve OS. With the limitations of a retrospective database study, the results of our study suggested that aggressive local therapy with RT followed by definitive surgery may provide patients the opportunity for improved long-term survival. Prospective studies examining the efficacy of RT on survival are necessary.

## Additional file

Additional file 1: Table S1. Comparison of patient, tumor and treatment factors for 5-year LFFS, DMFS and OS. **Table S2.** The subgroup analysis of the impact of RT on OS. (DOCX 27 kb)

## Abbreviations

CI: confidence interval
DMFS: distant metastasis-free survival
HR: hazard ratio
IMRT: intensity-modulated radiation therapy
LFFS: local failure-free survival
OS: overall survival
RT: radiotherapy
STS: soft tissue sarcoma
