# Supplementary material for: The efficacy of postoperative radiotherapy in localized primary soft tissue sarcoma treated with conservative surgery
Source: Radiat Oncol. 2016 Feb 25;11:25. doi: 10.1186/s13014-016-0605-y (PMC4766748; doi:10.1186/s13014-016-0605-y)
Supplement: Additional file 1: Table S1. — Comparison of patient, tumor and treatment factors for 5-year LFFS, DMFS and OS. Table S2. The subgroup analysis of the impact of RT on OS. (DOCX 27 kb) [file 13014_2016_605_MOESM1_ESM.docx]

Additional file 1

Table S1. Comparison of patient, tumor and treatment factors for 5-year LFFS, DMFS and OS

| Characteristic | LFFS | |  | DMFS | |  | OS | |
| --- | --- | --- | --- | --- | --- | --- | --- | --- |
|  | 5-year | *P* * |  | 5-year | *p* |  | 5-year | *p* |
| Age (years) |  | 0.700 |  |  | 0.626 |  |  | 0.745 |
| ≤50 | 68.8 |  |  | 76.0 |  |  | 72.5 |  |
| >50 | 71.3 |  |  | 77.2 |  |  | 69.9 |  |
| Gender |  | 0.510 |  |  | 0.786 |  |  | 0.735 |
| Male | 71.0 |  |  | 77.8 |  |  | 71.5 |  |
| Female | 68.8 |  |  | 77.0 |  |  | 70.8 |  |
| Location |  | 0.002 |  |  | 0.195 |  |  | 0.184 |
| Extremity | 73.2 |  |  | 77.8 |  |  | 77.9 |  |
| Trunk | 71.4 |  |  | 68.3 |  |  | 75.2 |  |
| Head/neck | 82.5 |  |  | 75.2 |  |  | 60.6 |  |
| Retroperitoneum | 52.5 |  |  | 90.0 |  |  | 65.7 |  |
| Histology |  | 0.970 |  |  | ＜0.001 |  |  | 0.012 |
| Rhabdomyosarcoma | 69.3 |  |  | 55.6 |  |  | 50.8 |  |
| Fibrosarcoma | 82.2 |  |  | 89.3 |  |  | 85.6 |  |
| MFH | 66.0 |  |  | 64.5 |  |  | 62.6 |  |
| Liposarcoma | 67.0 |  |  | 95.5 |  |  | 79.2 |  |
| Others | 73.1 |  |  | 73.3 |  |  | 73.9 |  |
| Tumor size (cm) |  | 0.006 |  |  | 0.405 |  |  | **0.005** |
| ≤5 | 80.3 |  |  | 79.8 |  |  | 80.2 |  |
| >5 | 71.0 |  |  | 76.0 |  |  | 66.0 |  |
| Grade |  | 0.438 |  |  | ＜0.001 |  |  | **0.022** |
| I | 83.2 |  |  | 96.4 |  |  | 91.9 |  |
| II | 71.4 |  |  | 92.2 |  |  | 78.7 |  |
| III | 66.1 |  |  | 67.0 |  |  | 64.0 |  |
| Margin status |  | 0.002 |  |  | 0.046 |  |  | ＜0.001 |
| Negative | 73.7 |  |  | 79.1 |  |  | 76.9 |  |
| Positive | 38.1 |  |  | 64.0 |  |  | 31.1 |  |
| Lymph node status |  | 0.647 |  |  | ＜0.001 |  |  | 0.010 |
| Negative | 69.9 |  |  | 79.4 |  |  | 72.9 |  |
| Positive | 78.8 |  |  | 40.0 |  |  | 40.0 |  |
| Chemotherapy |  | 0.508 |  |  | ＜0.001 |  |  | 0.005 |
| No | 69.2 |  |  | 81.2 |  |  | 74.3 |  |
| Yes | 73.3 |  |  | 61.4 |  |  | 58.1 |  |
| Radiotherapy |  | 0.004 |  |  | 0.307 |  |  | 0.089 |
| No | 63.6 |  |  | 80.8 |  |  | 65.0 |  |
| Yes | 81.1 |  |  | 73.1 |  |  | 74.8 |  |

Abbreviations: overall survival (OS); local failure-free survival (LFFS); distant metastasis-free survival (DMFS). *Determined by Log-rank test between adjacent categories.

Table S2. The subgroup analysis of the impact of RT on OS

| Characteristic | OS | | *P* |
| --- | --- | --- | --- |
|  | No-RT | RT |  |
| Age (years) |  |  | 0.473  0.535 |
| ≤50 | 66.8 | 77.9 |  |
| >50 | 69.7 | 69.8 |  |
| Gender |  |  | 0.938  0.195 |
| Male | 71.2 | 71.0 |  |
| Female | 65.9 | 78.0 |  |
| Location |  |  |  |
| Extremity | 71.8 | 83.0 | 0.142 |
| Trunk | 66.7 | 46.7 | 0.117 |
| Head/neck | 50.0 | 80.8 | 0.190 |
| Retroperitoneum | 70.1 | 50.0 | 0.350 |
| Histology |  |  |  |
| Rhabdomyosarcoma | 42.9 | 58.3 | 0.600 |
| Fibrosarcoma | 93.3 | 75.0 | 0.150 |
| MFH | 56.0 | 67.7 | 0.337 |
| Liposarcoma | 75.1 | 90.0 | 0.034 |
| Others | 66.7 | 80.8 | 0.631 |
| Tumor size (cm) |  |  |  |
| ≤5 | 74.6 | 86.7 | 0.381 |
| >5 | 66.2 | 65.9 | 0.585 |
| Grade |  |  |  |
| I | 90.3 | 100 | 0.309 |
| II | 75.0 | 85.7 | 0.197 |
| III | 60.8 | 67.4 | 0.445  81.5 |
| Margin status |  |  |  |
| Negative | 73.7 | 81.9 | 0.165 |
| Positive | 24.2 | 35.7 | 0.551 |
| Lymph node status |  |  |  |
| Negative | 70.6 | 75.8 | 0.325 |
| Positive | 25.0 | 50.0 | 0.400 |
| Chemotherapy |  |  |  |
| No | 72.1 | 77.6 | 0.312 |
| Yes | 51.5 | 63.6 | 0.309 |
